# Preventable pediatric hospitalizations and access to primary health care in Italy

**DOI:** 10.1371/journal.pone.0221852

**Published:** 2019-10-23

**Authors:** Rossella Zucco, Claudia Pileggi, Martina Vancheri, Rosa Papadopoli, Carmelo Giuseppe Angelo Nobile, Maria Pavia

**Affiliations:** 1 Department of Health Sciences, University of Catanzaro "Magna Græcia", Catanzaro, Italy; 2 Department of Pharmacy, Health and Nutritional Sciences, University of Calabria, Cosenza, Italy; Centre Hospitalier Universitaire Vaudois, FRANCE

## Abstract

The aim of this study was to quantify the burden of avoidable pediatric hospital admissions for Ambulatory care-sensitive conditions (ACSC) and to identify factors related to these preventable hospitalizations. The study was conducted by retrospectively reviewing all medical records of children admitted in a non-teaching 474-bed acute care hospital located in Catanzaro (Italy) for an avoidable hospitalization diagnosis. Two control clinical records involving children hospitalized for clinical conditions not classified as ACSC were randomly selected for each clinical record that included an ACSC. Among the 4293 pediatric hospitalizations, 451 (10.5%) were judged to be preventable. Of these, the most frequent discharge diagnoses were: dehydration (29.7%), pneumonia (17.7%), seizures (15.7%) and chronic obstructive pulmonary disease (12.9%).Children admitted for a preventable hospitalization were more likely to be females, to be younger, to be residents in the same province as the hospital and less likely to have had at least one Community-Based Pediatrician (CBP) access in the previous year and to have used the district health service. The burden of pediatric preventable hospitalizations found in this study is quite high, and the results show that there is still work that lies ahead on the way to improve interaction between hospital and community-based services.

## Introduction

Within the last few years there has been a radical change in the healthcare system in Italy, with calls for a more careful use of resources, and an in-depth evaluation of health care performance. In particular, great attention has been paid to community and primary care services, since a key objective in our health care system is to shift healthcare, whenever appropriate, from acute hospitals to community services [1].

This scenario has prompted the need for indicators measuring quality and performance of community services. “Ambulatory care sensitive conditions” (ACSC) are one well-used proxy measure for potentially preventable hospitalization[2]. More recently it has been suggested that rates of ACSC reflect quality of community-based care, rather than access, particularly in settings with universal health care [3–11]. Although variously defined, generally they are those conditions which respond well to interventions deliverable in community-based healthcare settings, and if managed can potentially prevent the need for hospitalization, or for which early intervention can prevent complications or more severe disease [2].

More recently, studies performed in the United States have shown the increasing costs generated by pediatric hospitalizations, highlighting the need for a careful monitoring activity even in this population[12–14].ACSCs have already been defined for children’s hospitalizations, including conditions whose onset can be prevented (such as through immunization), acute illnesses that could be controlled in ambulatory settings (such as a urinary tract infection), and chronic diseases that can be managed in outpatient settings (such as asthma)[5].

Very few studies performed in the United States and in some European countries have assessed pediatric hospital admission for ACSCs, reporting variable frequencies ranging from 5% to 43%[4,6,7,15]. However, Italian data are scanty and, when available, are related to certain specific diseases (eg, asthma, gastroenteritis)[8, 16,17].

The aims of this study were to quantify the burden of pediatric avoidable hospital admissions for ACSCs as an indirect measure of access to primary care services and to identify factors related to these preventable hospitalizations.

## Materials and methods

### Setting

The Italian National Health Service covers all citizens and legal foreign residents, with a universal coverage free of charge. The health districts are the operative branches of local health authorities and provide public health services and primary care including family medicine and community services. All residents are registered with a primary care physician (PCP) when they reach the age of 15 and, before this age, with a community based pediatrician (CBP). PCPs and CBPs provide various primary care services, such as health promotion and preventive activities, diagnosis, treatment, and follow-up of acute and chronic conditions. They also act as gate-keepers for access to secondary health services and for drug prescription for all patients on their list. These services are provided to all patients free of charge. Moreover, when the office of the PCP and CBP is closed Continuity of Care services provide night and weekend coverage as well as urgent home care to all patients. Community services also include home and residential care for the elderly and the disabled, and hospice care[1].

### Study population

This case-control study was conducted by retrospectively reviewing all medical records of patients under the age of 18, admitted between 1 January and 31 December 2016 to medical and surgical wards of a non-teaching acute care 474-bed hospital located in Catanzaro (Italy), for an avoidable hospitalization condition. The hospital included in the study was a general hospital with a catchment area from the entire Calabria region and a volume of pediatric admissions of numbering 4126 in 2015.

To identify the preventable hospitalizations from the administrative database, we used the list of 20 ambulatory care sensitive conditions (ACSCs) compiled by Casanova and Stratfield[5],each condition including a series of diagnostic sub-categories defined by diagnosis codes of the International Classification of Diseases (ICD-9-CM). For example, according to the Casanova and Stratfield classification, the definition of chronic obstructive pulmonary disease included chronic airway obstruction not elsewhere classified and bronchitis (Table 1). Admission for ACSCs were classified as preventable hospitalizations and considered as “cases”. All “cases” were judged to be appropriate hospitalization based on severity of illness but were considered potentially avoidable, since the underlying condition could have been adequately prevented or managed in the primary health setting. For each clinical condition including an ACSC, two control clinical records matched by year and month of admission and involving children admitted for clinical conditions not classified as ACSC were randomly selected. Children directly admitted to the pediatric intensive care unit were excluded. Only patients whose parents had given written permission for their personal data to be stored in the hospital database and used for research were included in the study.

**Table 1:** Frequency of specific preventable hospitalization conditions in the study population.

Condition	ICD9-CM Code		N	%
20. Dehydration-volume depletion	276.5	Disorders of fluid, electrolyte, and acid-base balance	134	29.7
9. Bacterial pneumonia	481	Pneumococcal Pneumonia	1	0.2
	486	Pneumonia, organism unspecified	79	17.5
8. Chronic obstructive pulmonary disease	496	Chronic airway obstruction, not elsewhere classified	1	0.2
	466.0	Bronchitis	57	12.6
3. Convulsions	780.3	Convulsions	47	10.4
2. Grand mal status and other epileptic convulsions	345	Epilepsy and recurrent seizures	23	5.1
4. Severe ear-nose-throat infections	382	Suppurative and unspecified otitis media	15	3.3
	463	Acute tonsillitis	2	0.4
	465	Acute upper respiratory infections of multiple or unspecified sites	29	6.4
10. Asthma	493	Asthma	23	5.1
18. Kidney/urinary tract infection	590	Infections of kidney	3	0.7
	599.0	Urinary tract infection, site not specified	9	2
	599.9	Unspecified disorder of urethra and urinary tract	1	0.2
15. Diabetes A, B, C	250.1	Diabetes with ketoacidosis	4	0.9
	250.0	Diabetes mellitus without mention of complication	7	1.5
17. Gastroenteritis	558.9	Other and unspecified noninfectious gastroenteritis and colitis	5	1.1
1.Immunizable-preventable conditions	033	Whooping cough	1	0.2
	052.0	Post varicella encephalitis	2	0.4
16. Hypoglycemia	251.2	Hypoglycemia, unspecified	2	0.4
14. Cellulitis	683	Acute lymphadenitis	1	0.2
12. Hypertension	401.0	Malignant	1	0.2
	401.9	Unspecified	1	0.2
21.Iron-deficiency anemia	280.9	Irondeficiency anemia, unspecified	2	0.4
Other conditions*			0	0
**Total**			**451**	

*Including: Pulmonary tuberculosis, Other tuberculosis, Congenital syphilis, Congestive heart failure, Angina, Dental conditions, Nutritional deficiencies, Failure to thrive, Pelvic inflammatory disease

Two physicians, who were not involved in patient care and who had previous training, collected the data by reviewing clinical charts and by phone interviewing parents of discharged patients.

The following data were collected for each patient by reviewing their medical charts: socio-demographics (child age and gender, parent’s marital status and education level); distance of patient's home from hospital; ward and type of admission; the healthcare figure that referred the patient to the hospital; primary and secondary diagnosis; and procedures and interventions performed during hospitalization. Moreover, a phone interview with parents of children included in the study was conducted to collect the following data: additional socio-demographic information (parents’ working activity and number of persons in the household); child health status; child’s utilization of health services during the previous year (number and main reasons for CBP visits or no CBP visits for medical control or consultation, difficulty of access to and satisfaction with CBP health services, number and main reasons for specialist visits, emergency accesses and previous hospital admissions); and the name of their own CBP. Questions on frequency of utilization of health services during the previous year were mostly in the "yes/no" format. If the answer was "yes", then the participants were asked about the number of accesses. The questions on satisfaction and difficulty with health services access were scored on a four point Likert scale with options for “no”, “few”, “rather”, and “much”. The questionnaire was pretested on a convenience sample of parents to ensure clarity of interpretation. A copy of the survey questions is reported in S1 Appendix.

To collect data from medical records, researchers were exempted from obtaining written consent by the patients who are requested during the hospitalization to give permission for their personal data to be used for research, as detailed by the Italian rules (Legislative Decree 101/2018). All study participants gave their verbal consent to participate in the telephone survey. Participation in the study was voluntary and anonymity and confidentiality issues were strictly observed. The study protocol was approved by the Institutional Ethical Committee (Ethical Committee of Calabria region-Central Area-15 June 2017).

### Statistical analysis

Stepwise multivariate logistic regression analyses were performed to identify baseline characteristics independently associated with the following outcomes of interest: preventable hospitalization for all the investigated conditions (No = 0; Yes = 1) (Model 1) and preventable hospitalization for dehydration (No = 0; Yes = 1) (Model 2).

The model building strategy included the following steps: univariate analysis of each variable considered, using the appropriate test statistic (chi-square test or *t* test); inclusion of any variable whose univariate test has a *P* value <0.25; ways to include independent variables in the model (continuous, ordinal or categorical) took into account how each of these ways better fitted the data at the univariate analysis and we chose the most appropriate way in the multivariate analysis. The explanatory variables included in the two models were the following: patient's age (continuous, in years); patient's sex (male = 0, female = 1); province of residence (other provinces = 0; Catanzaro = 1); additional children in the household (no = 0, yes = 1); CBP access in the previous year (No = 0, Yes, for a medical control or consultation = 1); CBP visit or telephone contact immediately before admission (No = 0, Yes = 1); district health services access in the previous year (No = 0, Yes = 1); parents’age (continuous, in years). The significance level for variables entering the logistic regression models was set at 0.2, and at0.4 for removing from the model. Adjusted odds ratio (ORs) and 95% confidence intervals (CIs) were calculated.

Stata version 14 statistical software package was used in collecting all data and conducting all data analysis [18].

## Results

Among the 4293 pediatric hospitalizations in the study period, 451 (10.5%) were judged to be preventable and included in the study. Among the remaining non preventable hospitalizations, 924 control clinical records were included in the study. All 1375 related parents were contacted for telephone interview. Of these, 170 did not complete the whole interview (43 cases and 127 controls), preferring not to answer some questions. The available data were however included in our analysis. (S2 Appendix). Dataset is available as Supporting information (S1 Database).

The main characteristics of the study population are presented in Table 2. Two hundred and thirty seven hospitalized children (19.7%) had chronic health conditions and 150 of these had been prescribed a chronic medication. Parents of these 150 children were asked if they had been adequately informed by the CBP about the correct way of drugs administration and doses and all respondents reported they were satisfied about the information provided by the CBP. The most frequent diagnoses were asthma (21.9%), neurological diseases (16.9%), congenital genetic disorders (11.4%), chronic inflammatory bowel disease (10.6%) and diabetes (8.4%).

**Table 2:** Selected characteristics of the study population according to type of hospitalization (preventable/non preventable).

Characteristics	Total N (%)*	Preventable hospitalization N (%)	Non preventable hospitalization N (%)
	1375	451	924
**Gender**			
Male	803 (58.4)	247 (30.8)	556 (69.2)
Female	572 (41.6)	204 (35.7)	368 (64.3)
		***p = 0*.*056***	
**Age, years (continuous)**			
Mean (±SD)	6.6 (5.6)	4.2 (4.5)	7.7 (5.7)
		***p<0*.*001***	
**Age group, years**			
<1	323 (23.5)	144 (44.6)	179 (55.4)
1–12	761 (55.3)	269 (35.3)	492 (64.7)
>12	291 (21.2)	38 (13.1)	253 (86.9)
Median	5	3	7
		***p<0*.*001***	
**Province**			
Catanzaro	1033 (75.1)	379 (36.7)	654 (63.3)
Cosenza	17 (1.2)	4 (23.5)	13 (76.5)
Crotone	154 (11.2)	24 (15.6)	130 (84.4)
Reggio Calabria	23 (1.7)	8 (34.8)	15 (65.2)
Vibo Valentia	95 (6.9)	25 (26.3)	70 (73.7)
Other	53 (3.9)	11 (20.7)	42 (79.3)
		***p<0*.*001***	
**Additional children in the household**			
None	370 (26.9)	131 (35.4)	239 (64.6)
1	593 (43.1)	207 (34.9)	386 (65.1)
>1	412 (30)	113 (27.4)	299 (72.6)
		***p = 0*.*021***	
**Ward of hospital stay**			
Pediatrics	1006 (73.2)	451 (44.8)	555 (55.2)
Pediatric surgery^#^	369 (26.8)	0 (-)	369 (100)
**Type of admission**			
Emergency	1262 (91.8)	451 (35.7)	811 (64.3)
Other	113 (8.2)	0 (-)	113 (100)
**Length of hospital stay, days**			
Mean (±SD)	3.8 (±3.1)	3.5 (±2.2)	4 (±3.4)
		***p = 0*.*008***	
**Type of discharge**			
Ordinary	1289 (93.7)	418 (32.4)	871 (67.6)
Voluntary/Other	86 (6.3)	33 (38.4)	53 (61.6)
		***p = 0*.*256***	
**CBP access in the previous year**			
None	511(42.4)	181 (35.4)	330 (64.6)
Yes	694 (57.6)	227 (32.7	467(67.3)
		***P = 0*.*126***	
**Satisfaction with CBP health services**			
No/few	73 (7.1)	31 (42.5)	42 (57.5)
Rather/much	948 (92.9)	355 (37.4)	593 (62.6)
		***p = 0*.*394***	
**Difficulty of access to CBP health services**			
No/few	957 (93.7)	361 (37.7)	596 (62.3)
Rather/much	64 (6.3)	25 (39.1)	39 (60.9)
		***p = 0*.*830***	
**Parent contacted CBP (by telephone or office visit) before admission**			
No	834 (69.2)	297 (35.6)	537 (64.4)
Yes	371 (30.8)	111 (29.9)	260 (70.1)
		***p = 0*.*054***	
**District health services access in the previous year**			
No	968 (80.4)	342 (35.3)	626 (64.7)
Yes	236 (19.6)	66 (28)	170 (72)
		***p = 0*.*032***	
**Satisfaction with district health services**			
No/few	6 (2.5)	1 (16.7)	5 (83.3)
Rather/much	230 (97.5)	65 (28.3)	165 (71.7)
		*p = 0*.*463*	
**Emergency access in the previous years**			
No	924 (76.7)	315 (34.1)	609 (65.9)
Yes	280 (23.3)	93 (33.2)	187 (66.8)
**Hospital admissions in the previous year**			
No	955 (79.3)	326 (34.1)	629 (65.9)
Yes	249 (20.7)	82 (32.9)	167 (67.1)
		***p = 0*.*721***	
**Chronic health condition**			
No	967 (80.3)	330 (34.1)	637 (65.9)
Yes	237 (19.7)	78 (32.9)	159 (67.1)
		***p = 0*.*723***	
**Medication for chronic health condition**			
No	1054 (87.5)	355 (33.7)	699 (66.3)
Yes	150 (12.5)	53 (35.3)	97 (64.7)
		***p = 0*.*689***	
**Parents’ education**			
Less than high school graduate	366 (31.4)	119 (32.5)	247 (67.5)
High school graduate or more	800 (68.6)	277 (34.6)	523 (65.4)
		***p = 0*.*480***	
**Parents’ age, years (continuous)**			
Mean (±SD)	39.1 (7.5)	36.6 (6.9)	40.3 (7.4)
		***p<0*.*001***	

* The numbers that do not add to 1375 are due to non-applicable data for the variable

CBP, Community-Based Pediatricians

^#^Including pediatric otolaryngology

Among the 451 preventable hospitalizations (Table 1), the most frequent discharge diagnoses were: dehydration (29.7%), pneumonia (17.7%), seizures (15.7%) and chronic obstructive pulmonary disease (12.8%). Moreover, 3 had a vaccine-preventable hospitalizations and 77.5% of respondents declared that their children had received only part of the recommended immunization schedule, while 11 (2.4%) parents were discouraged by healthcare professionals from having their children vaccinated.

Children admitted for a preventable hospitalization were more likely to be females compared to males (35.7% vs. 30.8%,p = 0.05), to be younger (mean 4.2 vs. 7.7 years old, p<0.001), to be residents in the same province as the hospital (Catanzaro) (36.7%, χ^2^ = 33.8, p<0.001), with the mean length of stay of 3.5 vs 4 days, p = 0.008). Moreover, they were less likely to have used the district health service in the previous year (28% vs. 35.3%, p = 0.032). Parents of the children admitted with avoidable hospitalization were significantly younger (mean age 36.6 vs. 40.3,p<0.001), did not have other children (35.4% vs. 31.8%,p = 0.021) and were less likely to have brought their children to a CBP for a medical consultation or to have spoken to the CBP on the telephone immediately before the hospitalization (35.6% vs. 29.9%,p = 0.054) (Table 2). For Model 1, the results of the multiple logistic regression analysis substantially confirmed the findings of the univariate analysis, except for the presence of additional children in the household. Moreover children admitted for a preventable hospitalization were less likely to have had CBP access for control or medical consultation in the previous year (Table 3).

**Table 3:** Multiple logistic regression analysis results.

Variable	OR	95% CI	P
***Model 1*: *Preventable hospitalization*** ***Log likelihood = -653*.*61; p<0*.*0001*, *No*. *of observations = 1155***			
**Age, years (continuous)**	0.86	0.83–0.89	<0.001
**Province**	1.00^a^		
Other	2.05	1.47–2.86	<0.001
Catanzaro			
**Parent contacted CBP**^**c**^ **before admission**			
No	1.00^a^		
Yes	0.67	0.50–0.91	0.010
**District health services access in the previous year**			
No	1.00^a^		
Yes	0.65	0.46–0.92	0.0217
**Parents’age, year (continuous)**	0.97	0.94–0.99	0.016
**CBP**^**b**^ **access in the previous year**			
**No**	1.00^a^		
**Yes**	0.49	0.36–0.65	<0.001
**Gender**			
Male	1.00^a^		
Female	1.25	0.95–1.64	0.104
**Additional children in the household**			
No	1.00^a^		
Yes	1.13	0.93–1.38	0.221
***Model 2*: *Preventable hospitalization for dehydration*** ***Log likelihood = -319*.*905; p<0*.*0001*, *No*. *of observations = 885***			
**Age, years (continuous)**	0.90	0.85–0.95	<0.001
**Province**			
**Other**	1.00^a^		
**Catanzaro**	2.17	1.28–3.70	0.004
**District health services access in the previous year**			
No	1.00^a^		
Yes	0.49	0.27–0.88	0.018
**Parent contacted CBP**^**b**^ **before admission**			
No	1.00^a^		
Yes	0.65	0.40–0.99	0.047
**Gender**			
Male	1.00^a^		
Female	1.24	0.82–1.87	0.290
**Parents’age, year (continuous)**	0.96	0.93–1.00	0.071
**Additional children in the household**			
No	^c^	^c^	^c^
Yes	^c^	^c^	^c^
**CBP**^**b**^ **access in the previous year**			
No	^c^	^c^	^c^
Yes	^c^	^c^	^c^

^a^ Reference category

^b^ Community-Based Pediatricians

^c^ Removed by the model

Univariate analysis for preventable hospitalization for dehydration showed the same results except for the presence of chronic diseases. Indeed, children admitted for dehydration were less likely to have chronic diseases (p = 0.006) (data not shown). Multivariate analysis substantially confirmed the findings of the univariate analysis (Table 3).

## Discussion

This study represents one of the first Italian attempts to quantify the proportion of pediatric avoidable hospital admissions for ACSCs, to evaluate the extent of the access to primary health care and to describe the relationship between the patient’s socio-demographic profile and health conditions and preventable hospitalizations. Although ACSCs have been less used in the pediatric population, the results of this study show the usefulness of these conditions as indicators of the burden of preventable hospitalizations. In this study context 10.5% of all pediatric hospitalizations were preventable; in previous studies performed in Europe and USA using the same definition of preventable hospitalization described in this study, prevalence of these conditions showed variable results ranging from 5% to 40%[6,7,19].Although high, our prevalence is one of the lowest when compared with the data reported in other studies[3,6,7].These data deserve to be highlighted, since they can reflect the quality of provision of outpatient care in the area of our study.

Four diagnoses (dehydration/gastroenteritis, pneumonia, seizures and chronic obstructive pulmonary disease) accounted for 90% of avoidable pediatric hospitalization, in line with similar studies in the literature[8, 20].In contrast to that reported in previous studies performed among the adult population[3,9], preventable hospitalizations for the pediatric population are more related to acute illnesses, highlighting some difficulties in managing acute diseases, which can often be solved if promptly treated at home, with CBP consultation and intervention. On the contrary, preventable hospitalizations for chronic diseases, such as asthma, appear to be lower compared with the findings of previous studies [4,10,20], pointing out a proper management of diagnostic-therapeutic pathways for chronic diseases in the area of our study. It should also be noted that the hospital included in the study was a general hospital and not just a pediatric hospital, where many of the hospitalizations are for children with chronic conditions.

Gastroenteritis and dehydration have already been reported as major sources of hospitalization in children, comprising almost 30% of all preventable hospitalizations, although a research carried out in 2010 showed a 64% reduction in hospitalization rates for this condition and this is the main reason why we also modeled eventual determinants of dehydration in our statistical analysis [15].This finding underscores the importance of timely and proper preventive measures for these conditions. Indeed, guidelines from the Centers for Disease Control recommend timely oral rehydration at home, early re-feeding, and avoidance of unnecessary medications [21],although it has been reported that CBPs and parents do not consistently follow these guidelines[22]. Hospitalization charges due to seizures and other convulsions accounted for 15.7% of all preventable hospital admission. The mechanisms for preventing hospitalizations due to seizures are likely similar to other conditions, including poor medication adherence[19,23]and, timely access to health care and evidence-based practices, but it should be highlighted that some (such as first-time) seizures cannot be controlled without hospitalization. Further research may be required to identify those hospital admissions that are actually preventable.

Two respiratory conditions, pneumonia and chronic obstructive pulmonary disease, comprised 30% of the potentially preventable pediatric hospitalizations in terms of total admissions. This finding underlines the importance of the adherence to clinical guidelines by the CBP. Indeed, recently published guidelines on treatment of community-acquired pneumonia addresses timely and appropriate outpatient management of bacterial pneumonia[24]. Also pneumococcal immunization rates can be associated with potentially preventable bacterial pneumonia[25, 26].

Younger children were more likely to be hospitalized for an ACSC and for dehydration. This finding has already been reported and in these studies younger children were also more likely to come to the hospital without a referral[6,19].It may be hypothesized that admitting doctors are more prone to hospitalize when the individual is an infant or young toddler, for which some clinical conditions may be more dangerous, such as, for example, a febrile neonate being at higher risk for serious bacterial infections than older children. Moreover, children with younger parents had a greater risk of an avoidable hospitalization or an admission for dehydration. In previous studies on avoidable acute respiratory diseases and diarrhea hospitalization, the Authors [27,28] found that children of young mothers have a greater risk of being hospitalized, probably because young parents are less prepared to prevent and manage these situations, which could otherwise be effectively solved according to the indications given by the CBP.

We did not find significant associations with other socio-economic conditions investigated, unlike other studies, that have highlighted, for example, for example how children living in areas of high income inequality have higher rates of hospitalizations for ACSCs[29], probably due to the universal nature of the Italian healthcare system.

Aligned with a previous study [20],our results indicate that preventable hospitalizations and admission for dehydration were more likely in those who had not contacted the CBP before hospitalization. Poor access to primary health care and lower adherence to the pediatric preventive care schedule may increase the likelihood of hospitalization for ACSCs. A related study on asthma hospitalizations identified that failure to contact the CBP before hospitalization was the greatest preventable risk factor for admission [10]. This finding highlights better outpatient primary care access and follow up as key approaches for reducing these hospitalizations. This conclusion has already been reached in several surveys conducted by some of us to assess access and satisfaction with primary care in our area which showed that many patients prefer to seek assistance at a more high level of care[30–33]

### Limitation

There are some potential limitations in the design and implementation of the study that should be considered when interpreting the results.

First, only principal diagnoses reported in the hospital discharge form according to the ICD-9 CM were included in our analysis. If reported secondary diagnoses were ACSCs, this could result in an underestimation of preventable hospitalizations. However, we presumed that hospital-based coding would be sufficient to accurately identify the most important cause for an admission.

Second, not all ACSC hospitalizations are preventable with proper primary care, depending on the severity of the presentation and underlying comorbidities. Thus, even with adequate primary care, there exists the possibility that children may be hospitalized for ACSC either because of extraneous factors or uncontrollable, fulminant exacerbation of their illness. Nevertheless ACSCs proved as useful indicators to measure the burden of preventable hospitalizations.

Third, we collected data also through a telephone interview. Self-reported responses, especially in relation to vaccinations received, can be influenced by the tendencies of participants to inaccurately or falsely answer the interviewer. Nevertheless, the parents were reassured that the data were analyzed in aggregate form.

Finally, we collected data from one hospital and concern about generalizability of our results may arise. However, although very scarce data is available in Italy on pediatric ACSCs, it seems, as for many other health services indicators, that there are differences between Northern and Southern Italy. In a recent report from the Observatory of the Health of the Italian Regions [34]pediatric avoidable hospitalization rates were similar in all Southern regions and consistently higher compared with Northern regions. For example, for gastroenteritis the preventable hospitalization rates in Southern Italy ranged from 2.41 discharges/1.000 inhabitants in Campania to 3.61 discharges/1.000 inhabitants in Apulia (Calabria region has a rate of 2.08 discharges/ 1.000 inhabitants). In the north of Italy rates ranged from 0.29 discharges/1.000 inhabitants in Valle d'Aosta to 1.36 discharges/1.000 inhabitants in Bozen's autonomous administration, whereas the overall national rate is 2.19 discharges/1.000 inhabitants [34]. Therefore, although we cannot exclude that our results pertain only to our area, we are confident that the findings of the study may be representative at least of the Southern Italian regions, even more considering that in Southern Italy there are only three pediatric hospitals, which are not teaching hospitals. To have more insight into provision of primary care in other areas as measured by avoidable hospitalization, we strongly suggest replication of the study in other regions of the country.

## Conclusion

The burden of pediatric preventable hospitalizations found in our study is quite high and the results show that there is still substantial work that lies ahead on the way to improve interaction between hospital and community-based services. Efforts are still needed and should focus on developing and implementing interventions to improve delivery of health care at the community level. Moreover, results show that acute illness management might benefit from effective physician- patient communication and parent education.

## Supporting information

S1 Appendix Survey instrument. (DOCX)

S2 Appendix Flow chart describing the study population. (DOCX)

S1 Database (XLSX)

## Abbreviations

ACSC: Ambulatory Care-Sensitive Conditions
CBP: Community-Based Pediatricians
