# Supplementary figures and images for: Preventable pediatric hospitalizations and access to primary health care in Italy
Source: PLoS One. 2019 Oct 23;14(10):e0221852. doi: 10.1371/journal.pone.0221852 (PMC6808327; doi:10.1371/journal.pone.0221852)

Appendix 1. Survey instrument


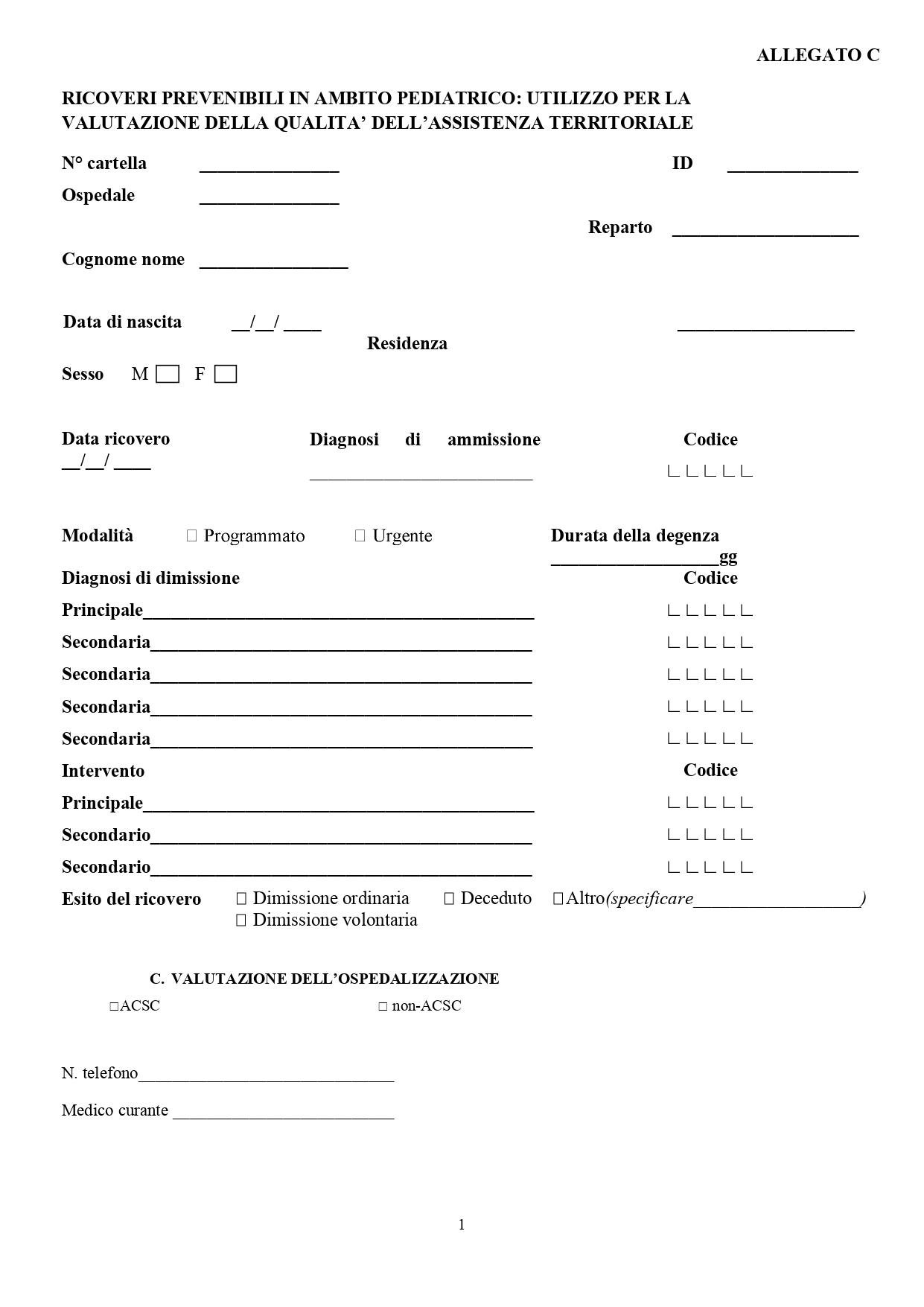


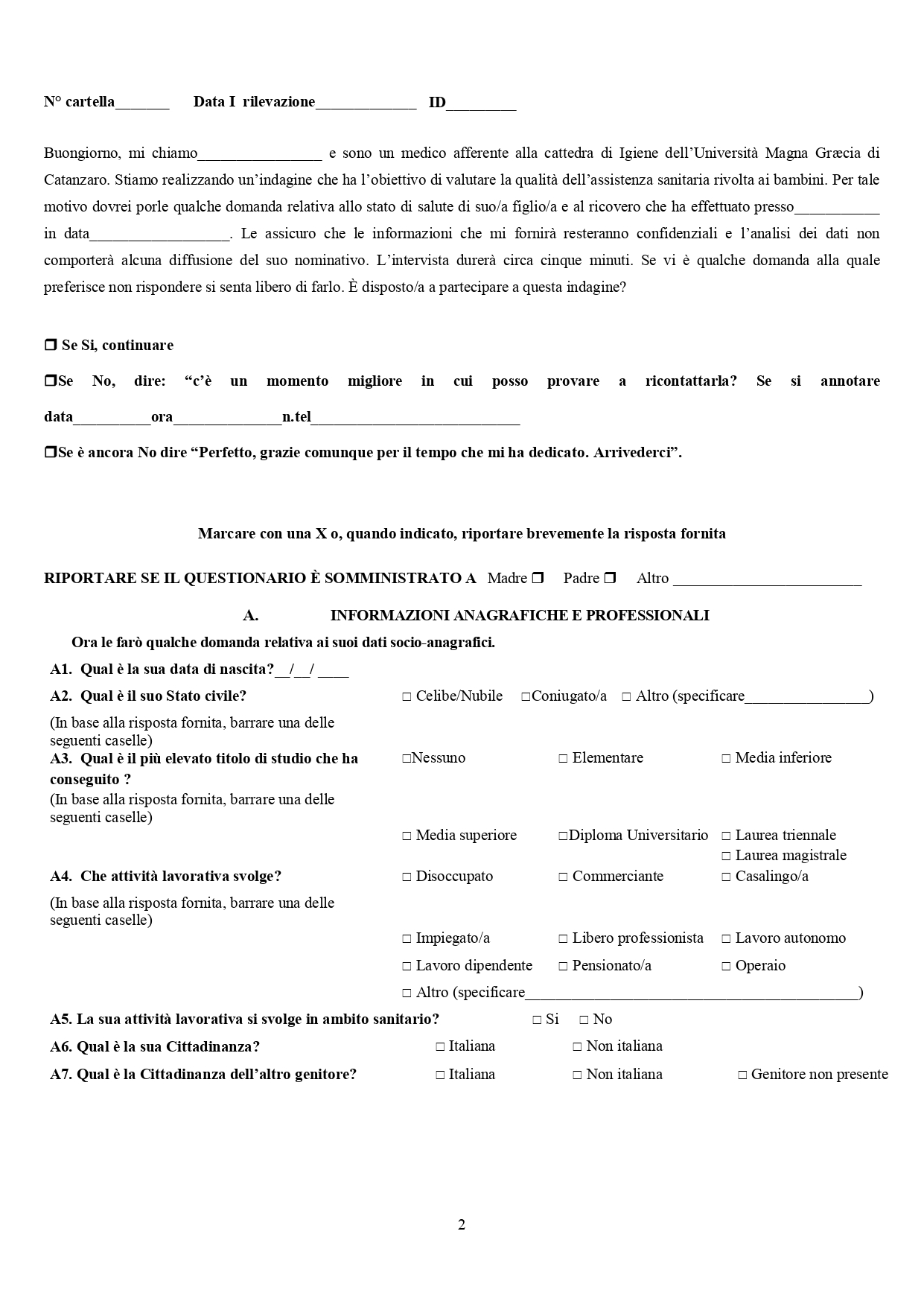


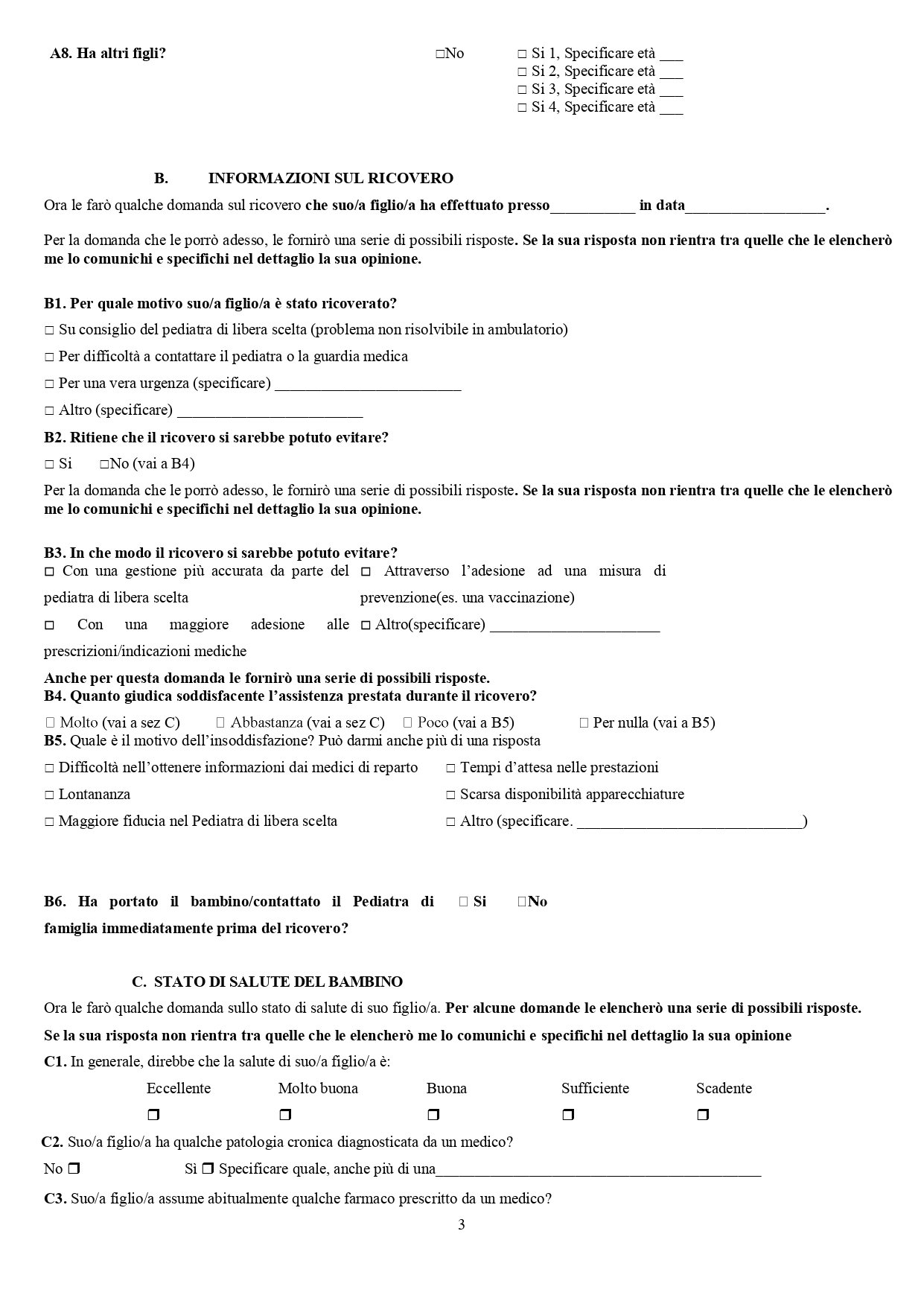


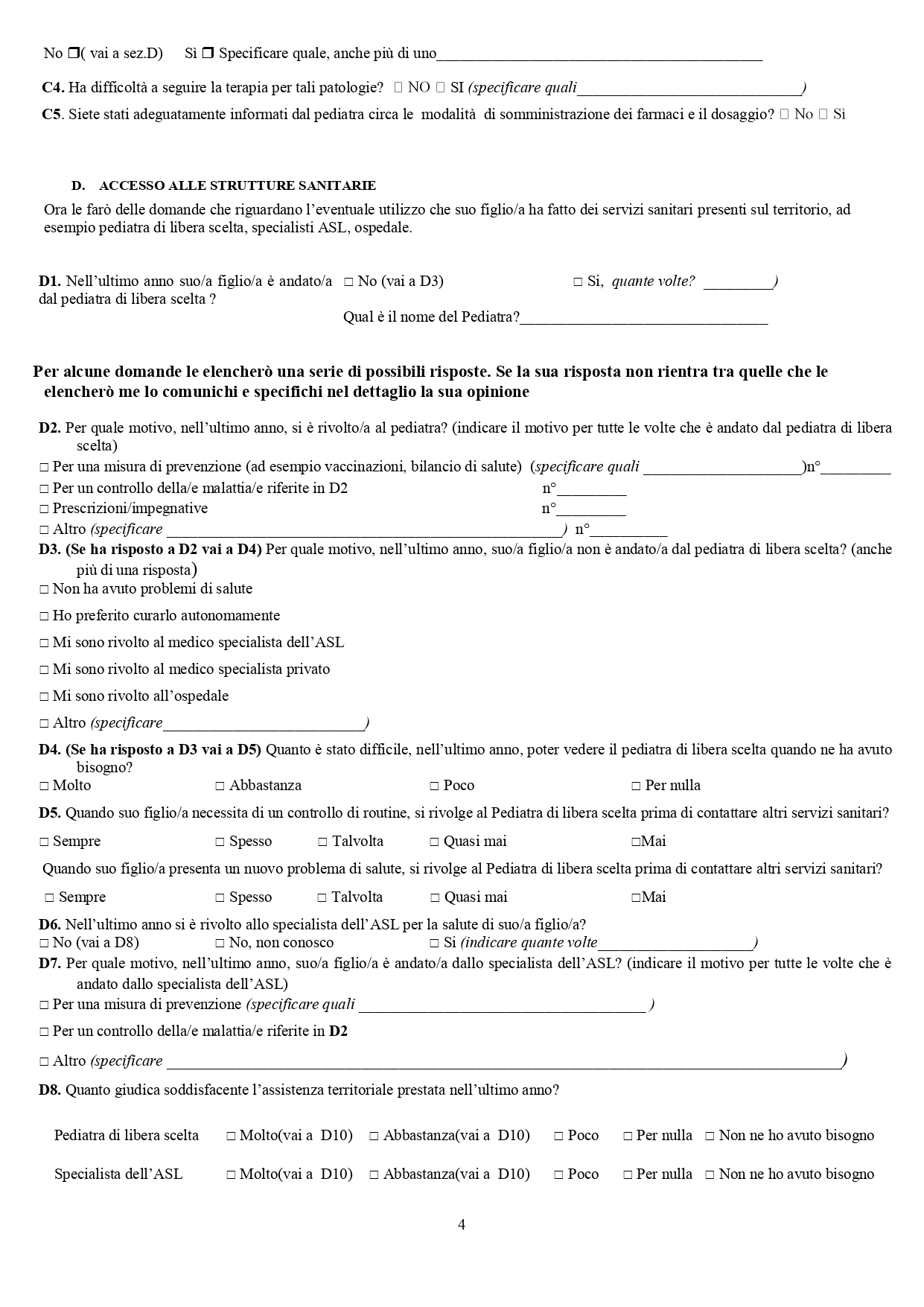


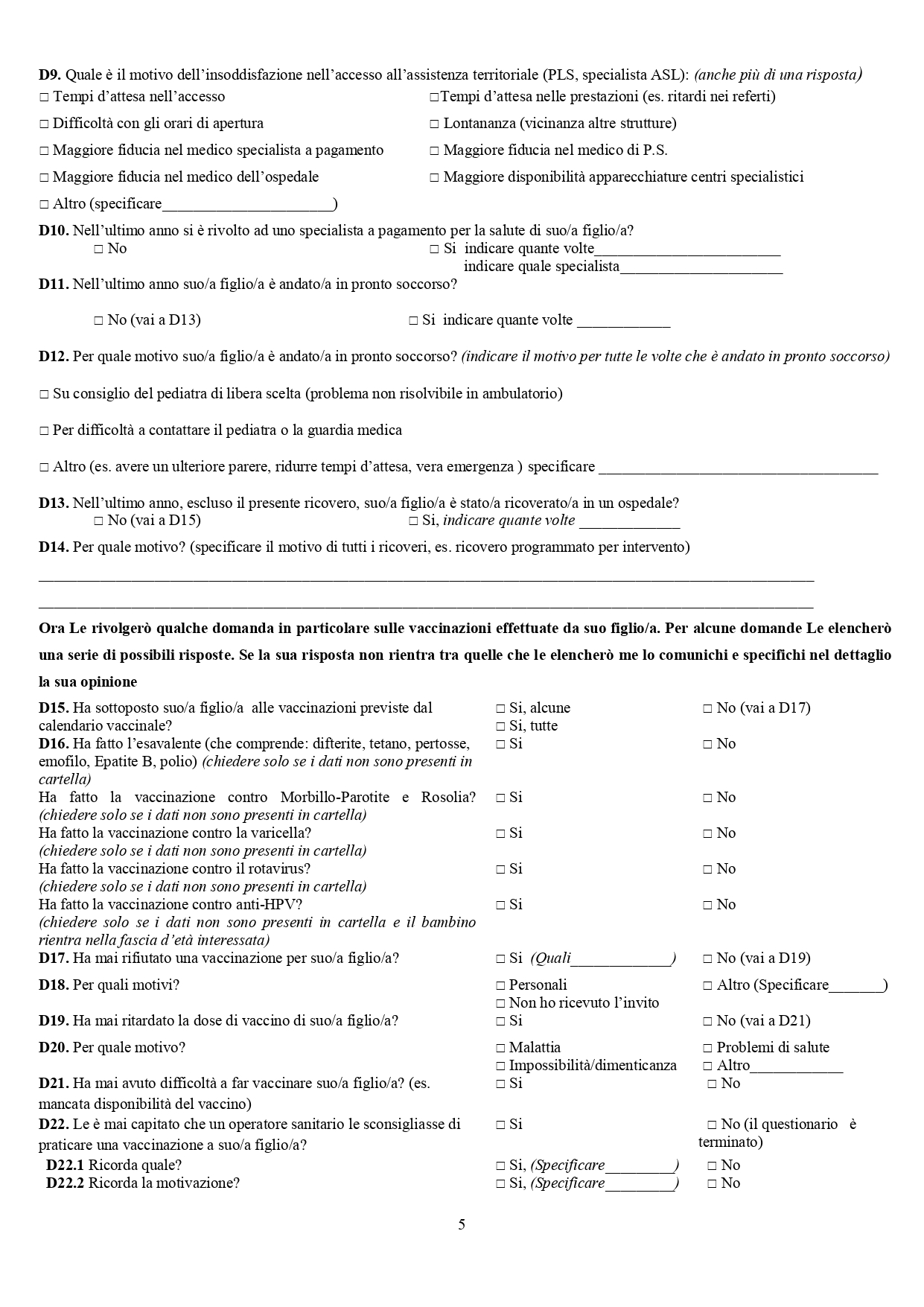


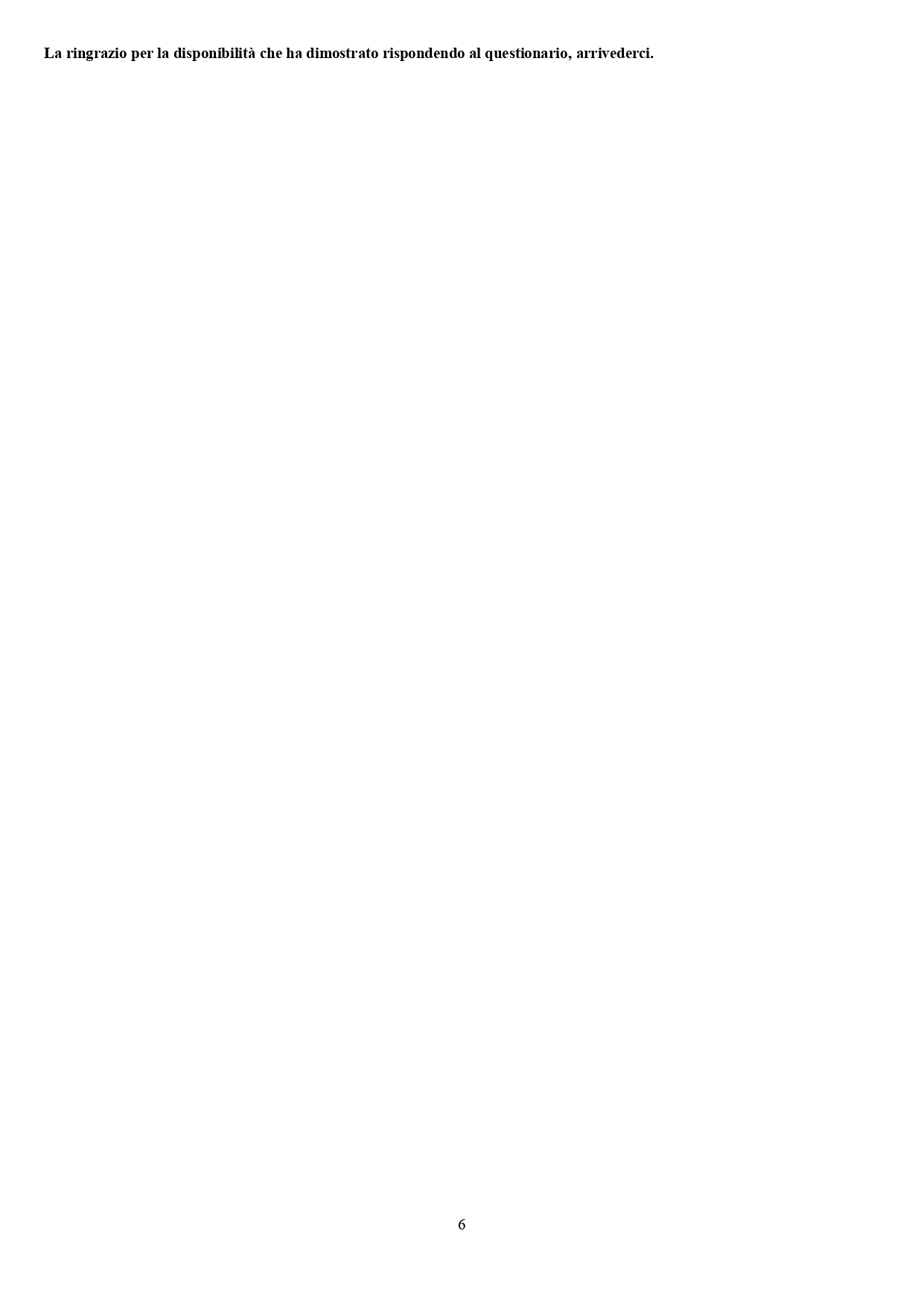


**
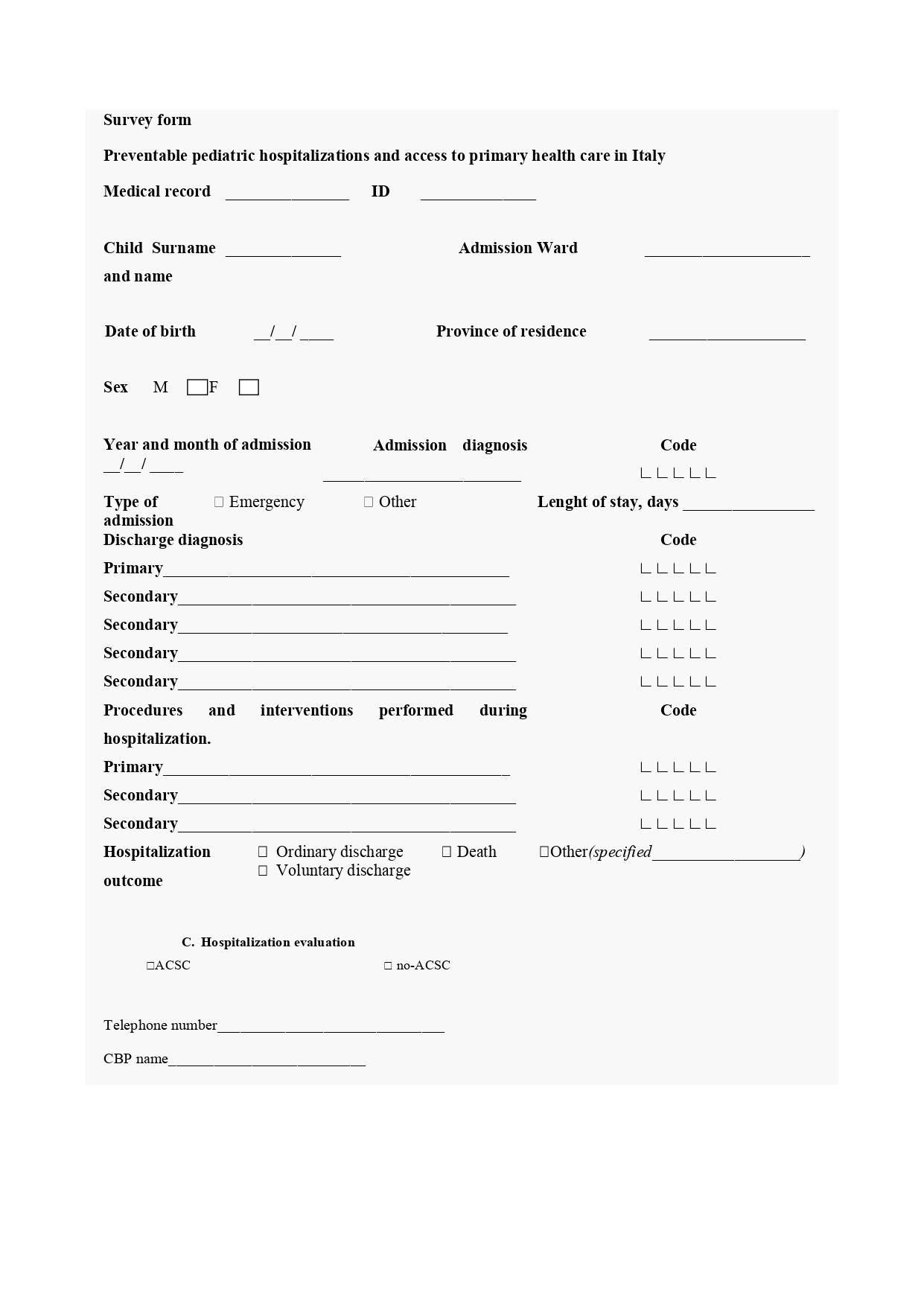
**


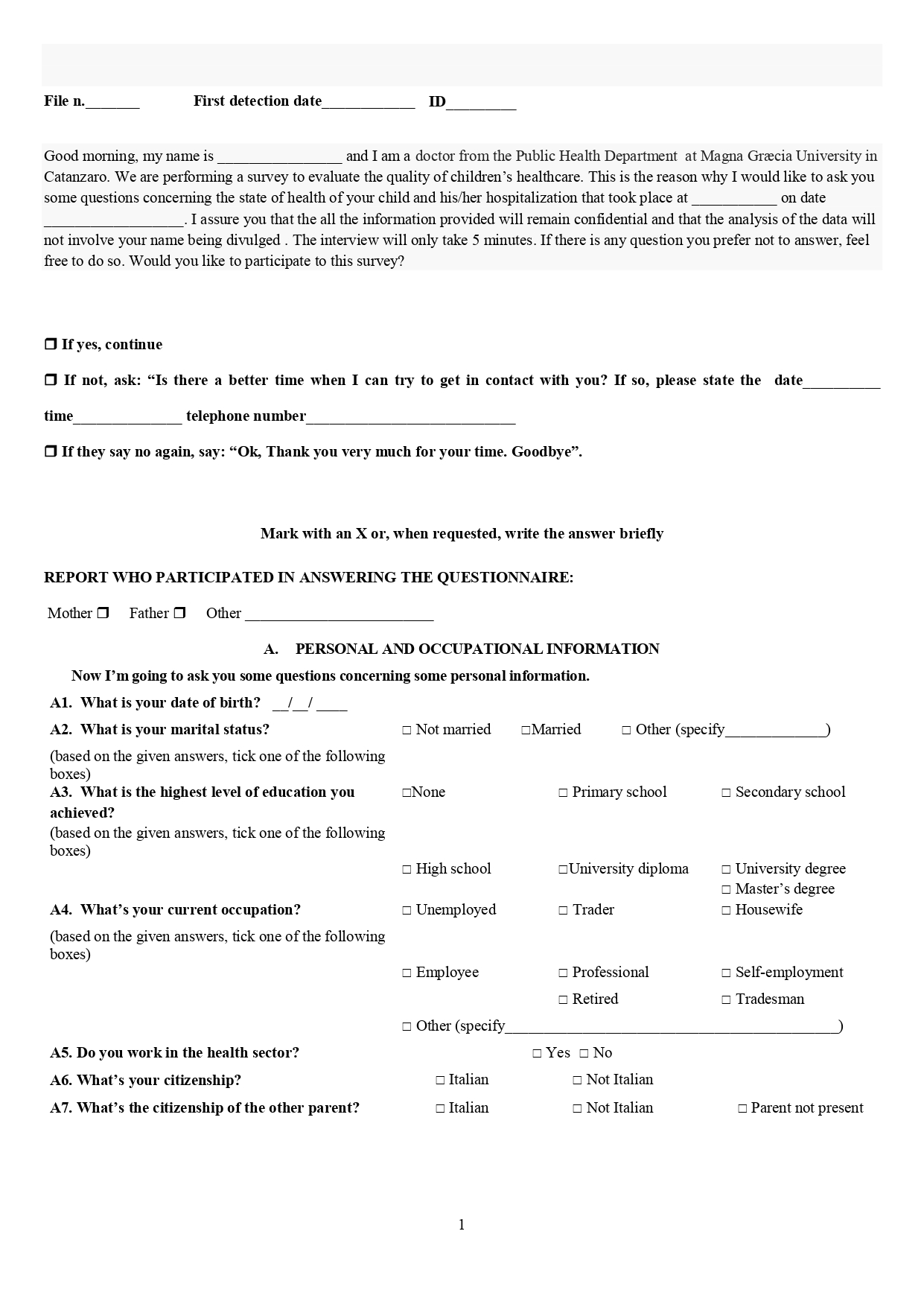


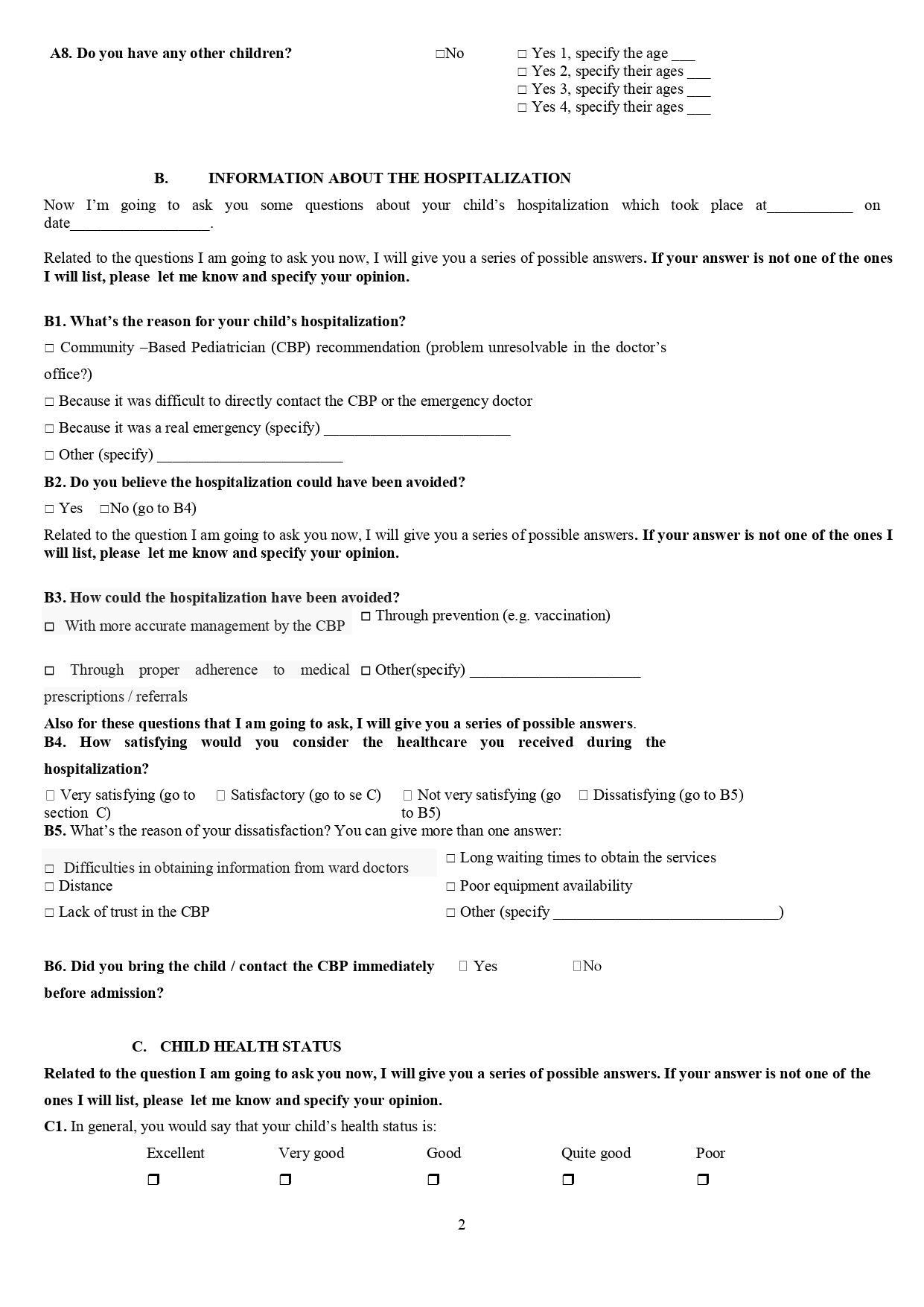


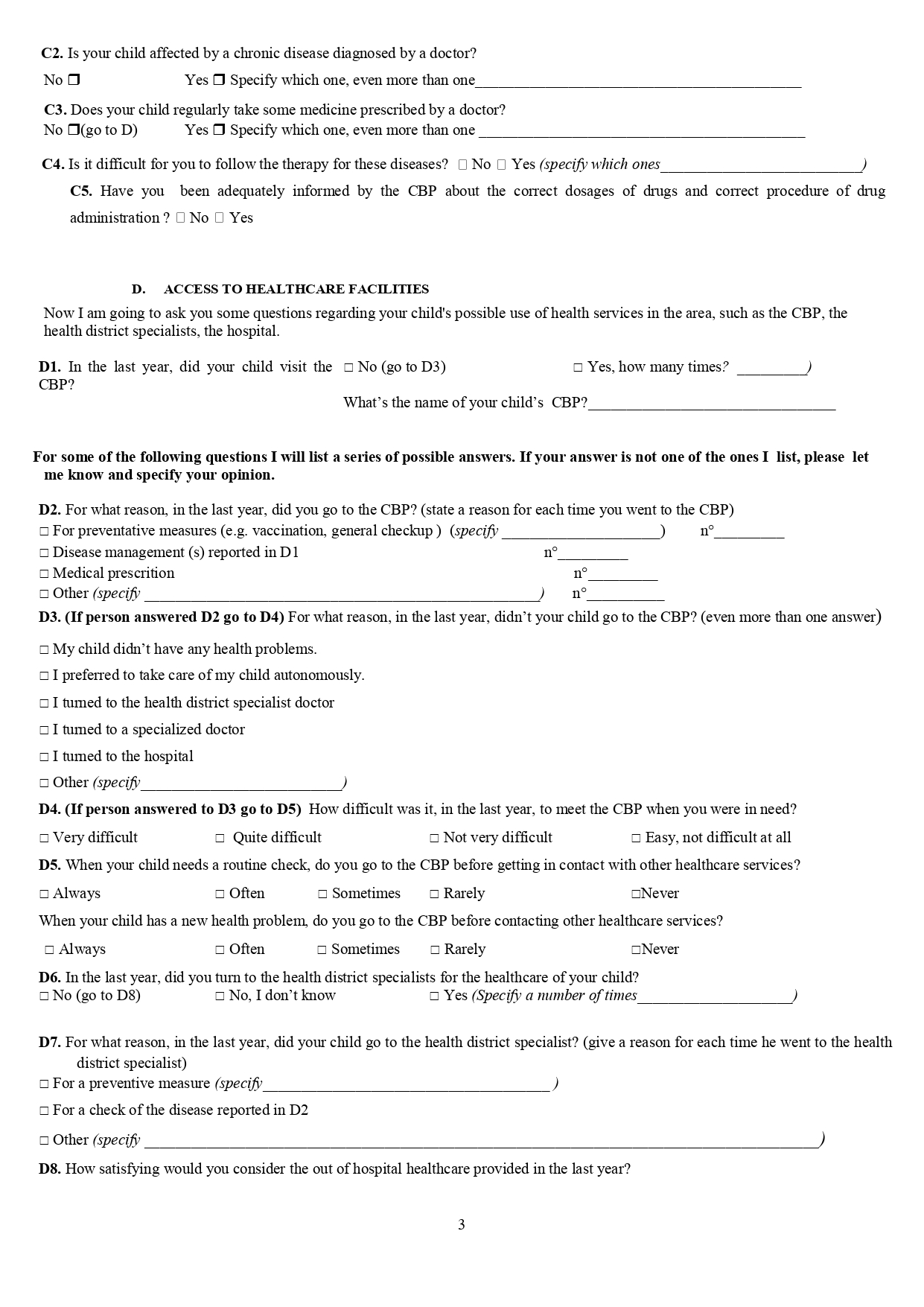


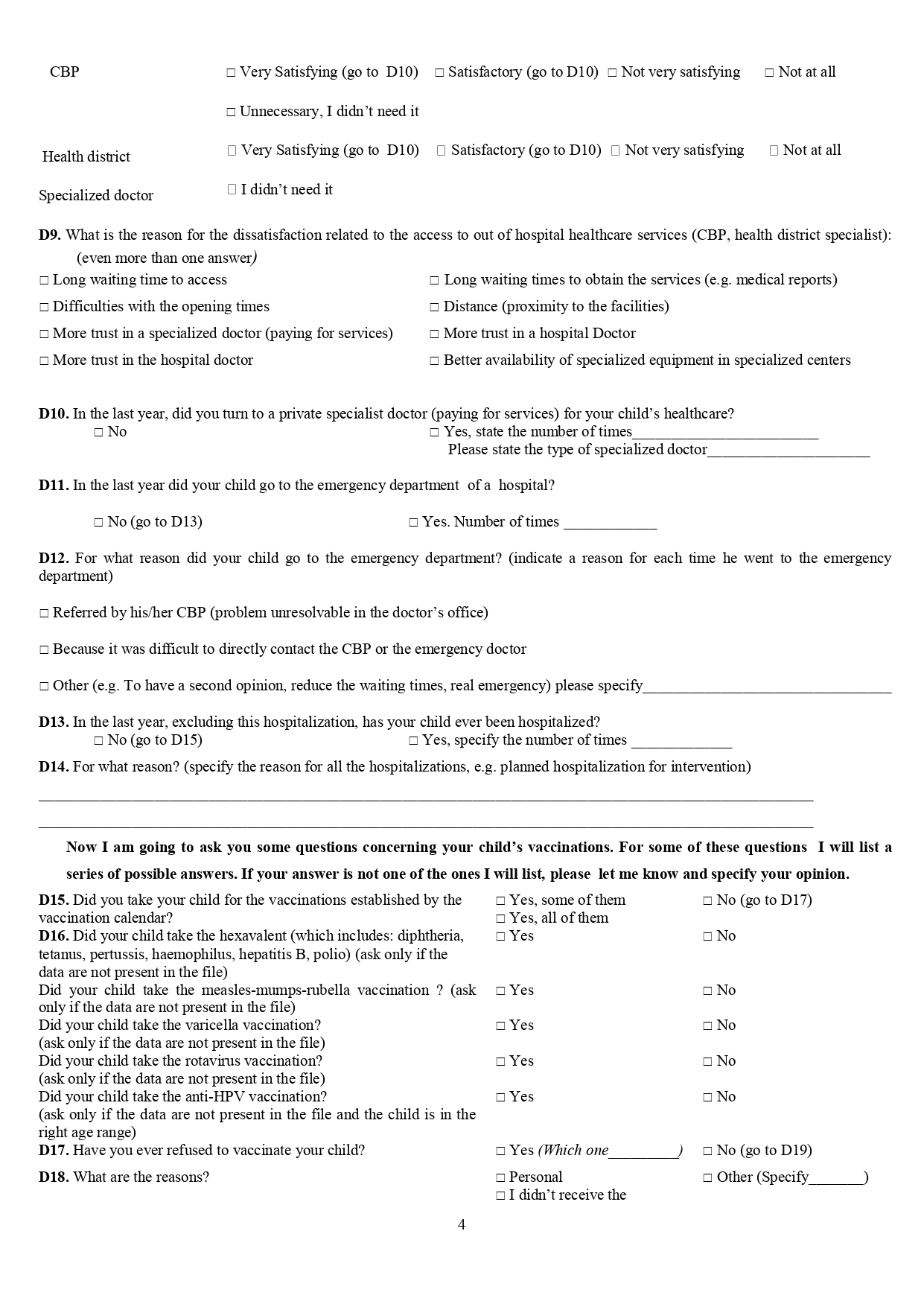


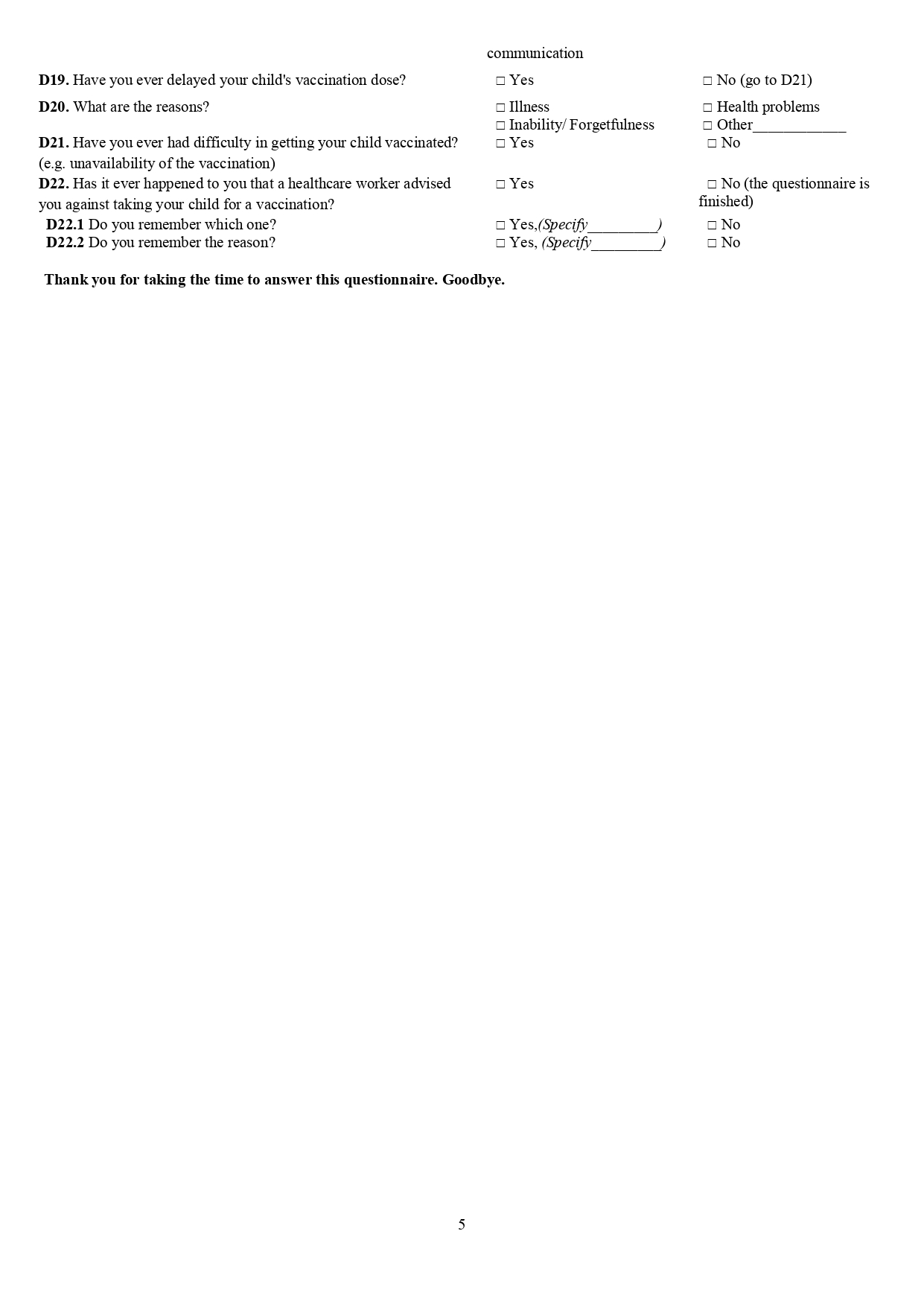

Supplement: S1 Appendix — (DOCX) [file pone.0221852.s001.docx]
