# Supplementary material for: Preventable pediatric hospitalizations and access to primary health care in Italy
Source: PLoS One. 2019 Oct 23;14(10):e0221852. doi: 10.1371/journal.pone.0221852 (PMC6808327; doi:10.1371/journal.pone.0221852)
Supplement: S2 Appendix — (DOCX) [file pone.0221852.s002.docx]

**APPENDIX 2. Flow chart describing the study population**

PREVENTABLE* HOSPITALIZATION

N= 451

NON PREVENTABLE* HOSPITALIZATION

N= 924

STUDY POPULATION

N=1375

NON PREVENTABLE HOSPITALIZATION

N=924

PREVENTABLE HOSPITALIZATION

N= 451

COMPLETE INTERVIEW

N=408

COMPLETE INTERVIEW

N=797

INCOMPLETE INTERVIEW

N=43

INCOMPLETE INTERVIEW

N=127

*Matched by year and month of admission
